# Treatment Delays and Survival Divides: Race, Sex, and Early-Onset Colorectal Cancer Disparities

**DOI:** 10.1158/2767-9764.CRC-25-0659

**Published:** 2026-01-29

**Authors:** Meng-Han Tsai, Jorge E. Cortes, Minjee Lee, Humberto Sifuentes, Sejong Bae

**Affiliations:** 1 Georgia Prevention Institute, Augusta University, Augusta, Georgia.; 2 Georgia Cancer Center, Augusta University, Augusta, Georgia.; 3 Department of Population Science and Policy, Southern Illinois University School of Medicine, Springfield, Illinois.; 4 Department of Gastroenterology and Hepatology, Augusta University, Augusta, Georgia.; 5 Department of Biostatistics, Data Science and Epidemiology, School of Public Health, Augusta University, Augusta, Georgia.

## Abstract

Our study examined the association between sex, race/ethnicity, and treatment timeliness with cause-specific survival (CSS) and overall survival (OS) among patients with early-onset colorectal cancer (EOCRC; diagnosed <50 years of age). We conducted a retrospective cohort analysis using data from the 2006 to 2020 Surveillance, Epidemiology, and End Results Program. Exposures were sex, race/ethnicity [non-Hispanic White (NHW), non-Hispanic Black (NHB), non-Hispanic Asian/Pacific Islanders (Asian/PI), non-Hispanic American Indian/Alaskan Native (AI/AN), and Hispanic], and treatment timeliness. Outcomes were CSS for colorectal cancer death and OS for any causes of death. Cox proportional models were applied. Among 79,090 patients with EOCRC, females (73.1% vs. 72.8% for males) were more likely to receive timely treatment and males had higher proportion of delay (7.5% vs. 6.9% for females; *P* < 0.001). Racial minorities had significantly greater delays (AI/AN 5.3%, Hispanic 4.2%, NHB 3.4%, Asian/PI 3.1% vs. NHW 2.2%; *P* < 0.05). Adjusted Cox models showed elevated colorectal cancer and overall mortality among males (14%–18% for CSS and 15%–20% for OS; *P* < 0.05) and NHB patients (23%–37% for CSS and 21%–34% for OS; *P* < 0.05) compared with females and NHW patients, respectively. Hispanic patients with no/unknown treatment had 22% higher colorectal cancer mortality [95% confidence interval (CI), 1.03–1.44] and 23% higher overall mortality (95% CI, 1.06–1.44). AI/AN/Asian/PI individuals had a 19% increased risk of overall mortality with delayed treatment (95% CI, 1–1.42). In conclusions, male and NHB patients had poorer CSS and OS regardless of treatment timing. Hispanic/AI/AN/Asian/PI patients faced higher mortality with delayed/undocumented treatment timing.

**Significance:**

Targeted strategies should focus on improving timely access to high quality of care for AI/AN/Asian/PI patients while also addressing barriers beyond treatment timeliness, particularly for male and NHB patients.

## Introduction

Early-onset colorectal cancer (EOCRC), defined as colorectal cancer diagnosed in individuals under 50 years of age, has been increasing in incidence in the United States (1). Compared with those diagnosed with average-onset colorectal cancer (age 50 years and older), adults with EOCRC are more likely to experience delays in diagnosis (2) and present with more advanced stages of disease, likely because detection is driven by symptoms (3–5). These diagnostic delays may occur because most patients with EOCRC are not eligible for routine screening, which can lead to delayed access to care (6) and, consequently, poorer survival outcomes (7). Therefore, understanding the patterns of timely access to care, such as time to treatment initiation following a cancer diagnosis, is critical as improving this process could significantly enhance outcomes for patients with colorectal cancer (8).

Although there is no standardized definition of what constitutes “timely” treatment, several studies, although using different definitions of timeliness, have demonstrated the impact of receiving timely treatment on survival. For example, the risk of mortality increases progressively with treatment delays—increasing by approximately 12% when treatment is delayed beyond 4 weeks, 24% beyond 8 weeks, and 39% beyond 12 weeks after diagnosis, compared with treatment initiated within 4 weeks of diagnosis (9). Cone and colleagues also found that longer time to treatment initiation was associated with worse survival for stage III colon cancer, with 5-year predicted mortality increasing from 38.9% (time to treatment, 61–120 days) to 47.8% (time to treatment, 181–365 days). Survival was better for those diagnosed with cancer at earlier stage (stage I and II; ref. 8). The start of adjuvant chemotherapy from surgery greater than 6 weeks after surgery was also associated with a 24% decline in survival probability for patients with colorectal cancer (10).

Demographic disparities were also evident in the impact of timely treatment on survival. Male patients with EOCRC experienced a longer delay before receiving their first treatment compared with female patients (27.6 days vs. 25.7 days; ref. 11). Another study found that Black and Hispanic adults experienced significantly more barriers (e.g., long waiting times and lack of transportation) to timely medical care than White adults (12). Importantly, young individuals from racial minority groups, such as American Indian or Alaska Native, Black, and Native Hawaiian or other Pacific Islander populations, were more likely to be diagnosed with cancer, including colorectal cancer, at a later stage and experience poorer survival outcomes (6, 13). These disparities are largely attributed to limited access to timely care, which is often exacerbated by socioeconomic disadvantages and structural barriers faced by these underserved populations (7, 14). This evidence underscores the complex interplay between demographic factors and the timeliness of treatment in shaping survival outcomes for patients with colorectal cancer.

Although a few studies have explored sex or racial disparities in EOCRC survival individually (15–17), to our knowledge, none has examined the combined influence of demographic subgroups and treatment timeliness. There remains a significant gap in integrated research that specifically identifies which demographic subgroups are most affected by delays in treatment and their impact on EOCRC mortality. Only one study highlighted that racial disparities persist in access to timely treatment for patients with EOCRC focusing on socioeconomic and structural challenges but without examining specific demographic groups (14). Thus, we investigated the relationship between sex, race/ethnicity, and timeliness of treatment (e.g., timely, delayed, severely delayed, and no/unknown) with both cause-specific survival (CSS) for colorectal cancer and overall survival (OS) at 5-year intervals. This study addresses a critical gap by leveraging nationally representative data to inform strategies that promote equitable and timely cancer treatment for young patients. Such insights are crucial for understanding the drivers of survival differences and informing targeted interventions, particularly among young populations who may face additional barriers by being outside eligibility thresholds for current screening recommendations.

## Materials and Methods

### Study design

We conducted a retrospective cohort analysis using data from the 2006 to 2020 Incidence Data with Census Tract Attributes from the Surveillance, Epidemiology, and End Results (SEER) Program (November 2022 submission). This specialized dataset includes 22 cancer registries, excluding Alaska and Illinois. These comprehensive, population-based US data include patient demographics, tumor site and morphology, stage at diagnosis, initial treatment, follow-up for vital status, and census tract–level socioeconomic indicators such as the Urban–Rural Indicator Code (URIC) and socioeconomic status (SES) quintile. The study population comprised patients diagnosed with colorectal cancer, defined by the SEER Site Recode International Classification of Diseases for Oncology-3/World Health Organization 2008 definition of colon cancer (C180–C189), rectosigmoid junction cancer (C199), and rectal cancer (C209). Appendix cancers were excluded in accordance with recent colorectal cancer reclassification guidelines (18). Data extracted for this study were publicly available and deidentified and thus considered exempt from institutional review board review at Augusta University.

### Study participants

A total of 835,907 patients with colorectal cancer were identified in the 2006 to 2020 SEER database. We first excluded duplicate records for the same patients (*n* = 11,338). To obtain the eligible study sample for patients with EOCRC, we excluded patients with colorectal cancer aged <20 years or aged 50 years or older or with unknown age information (*n* = 729,234) and those with colorectal cancer located in the appendix (*n* = 8,995). Furthermore, we excluded patients with survival time less than 30 days who lacked treatment time data (*n* = 4,832), unknown race (*n* = 668), unknown primary site (*n* = 45), or unknown SES quintile (*n* = 1,705). As a result, 79,090 eligible patients with EOCRC were included in the full analysis (Supplementary Fig. S1).

### Measures: outcome, exposure, and covariates

CSS for colorectal cancer death and OS for death due to any causes at 5-year intervals were outcomes of interest. Sex (male or female) and race/ethnicity [non-Hispanic White (NHW), non-Hispanic Black (NHB), non-Hispanic Asian/Pacific Islanders (Asian/PI), non-Hispanic American Indian/Alaskan Native (AI/AN), and Hispanic], and timeliness of treatment were exposures of interest. For treatment timeliness, we considered initiation within 1 month as timely, between 1 and 2 months as delayed, beyond 3 months as severely delayed, and cases lacking treatment timing data as no/unknown. In the absence of a standard definition for treatment timeliness in the literature, we adopted this categorization based on studies indicating increased mortality risk when treatment is delayed beyond 4 weeks (9). Furthermore, when treatment timing is not recorded in SEER, it is unclear whether the patient did not receive treatment or whether the registry failed to capture it. Because we could not distinguish between truly missing treatment timing and cases for which treatment was not received, we classified these as one group labeled “no/unknown”.

Covariates of interest included age at diagnosis (20–29, 30–39, and 40–49 years), marital status (married, unmarried, or unknown), four-level rurality, and census tract SES level (low, medium, and high). The definition of rurality is based on the URIC and takes into account the U.S. Census Bureau’s percentage of the population living in nonurban areas defined by four categories: 100% urban (all urban), ≥50% but <100% urban (mostly urban), >0% but <50% urban (mostly rural), and 100% rural (all rural) tracts. For tumor characteristics, we included stage at diagnosis (localized, regionalized, distant, or unknown), tumor grade (well differentiated, moderately differentiated, poorly differentiated, undifferentiated, or unknown), and primary site (right or left). Primary site was categorized as right colon (cecum through transverse colon) and left colon (splenic flexure through rectum).The selection of these variables was informed by prior literature indicating their potential impact on EOCRC survival (6). Finally, we grouped the year of diagnosis (2006–2010, 2011–2015, and 2016–2020) as one of the covariates to address changes in colorectal cancer treatment over time.

### Statistical analysis

Descriptive statistics were used to describe the distribution of patients with EOCRC according to sex, race/ethnicity, timeliness of treatment, sociodemographic characteristics, tumor characteristics, and year of diagnosis. Bivariate differences in the relationship of timeliness of treatment with sex, race/ethnicity, sociodemographic characteristics, tumor characteristics, and year of diagnosis were examined using *χ*^2^ tests. Kaplan–Meier survival curves and log-rank tests were used to compare CSS and OS across sex and race/ethnicity, stratified by timeliness of treatment. Survival time was measured in months, with all patients followed for up to 60 months. A 60-month (5-year) interval was used as it represents the standard time frame for evaluating survival outcomes. For CSS, patients who died from causes unrelated to colorectal cancer were considered censored. For OS, patients who were alive at the end of follow-up or lost to follow-up were considered censored. Furthermore, we performed multivariable Cox proportional hazards (PH) models to examine the association between sex, race/ethnicity, timeliness of treatment, and CSS for colorectal cancer and OS. To account for competing events, we also applied competing risk models to confirm these associations for colorectal cancer–specific survival, and as such, models provide estimates of the cumulative incidence of the event. Competing risk models provide less biased estimates of colorectal cancer–specific mortality by accounting for deaths from other causes, offering a clinically relevant measure and preventing overestimation of associations. Base and adjusted models were performed. The base model included all exposures (sex, race/ethnicity, and timeliness of treatment), whereas the adjusted model further adjusted for other sociodemographic characteristics (age at diagnosis, marital status, SES level, and rurality), tumor characteristics (stage at diagnosis, tumor grade, and primary site), and year of diagnosis. We also assessed interactions between sex and treatment timeliness, as well as race/ethnicity and treatment timeliness for base and adjusted models. This approach allows us to assess potential effect modification by treatment timeliness. Stratified analyses were conducted to examine sex and racial/ethnic differences on CSS and OS by timeliness of treatment. AI/AN/Asian/PI patients were combined in models because of small sample size for stratified analyses, particular for AI/AN patients (see Table 1). Lastly, we conducted additional analyses excluding individuals with missing or unknown treatment timing data to assess differences in demographic characteristics and their association with mortality. For models, adjusted hazard ratios (HR) associated with 95% confidence interval (CI) were calculated. The level of statistical significance was set at an α level of 0.05 and the *P* values were based on two-sided tests. We used SAS version 9.4, SAS Institute Inc., to perform all the analyses.

**Table 1.** Characteristics of patients with EOCRC by timeliness of treatment.

Characteristic	Total (*n* = 79,090)	Timely (*n* = 57,676, 72.9%)	Delayed (*n* = 5,725, 7.2%)	Severely delayed (*n* = 2,281, 2.9%)	No/unknown (*n* = 13,408, 17%)	*P* value
Sex	​	​	​	​	​	<0.001
Female	36,998 (46.8%)	27,054 (73.1%)	2,569 (6.9%)	968 (2.6%)	6,407 (17.3%)	​
Male	42,092 (53.2%)	30,622 (72.8%)	3,156 (7.5%)	1,313 (3.1%)	7,001 (16.6%)	​
Race	​	​	​	​	​	<0.001
NHW	43,283 (54.7%)	32,085 (74.1%)	2,817 (6.5%)	933 (2.2%)	7,448 (17.2%)	​
NHB	10,915 (13.8%)	7,727 (70.8%)	734 (6.7%)	375 (3.4%)	2,079 (19.1%)	​
Hispanic	17,245 (21.8%)	12,366 (71.7%)	1,552 (9%)	723 (4.2%)	2,604 (15.1%)	​
AI/AN	510 (0.6%)	385 (75.5%)	45 (8.8%)	27 (5.3%)	53 (10.4%)	​
Asian/PI	7,137 (9%)	5,113 (71.6%)	577 (8.1%)	223 (3.1%)	1,224 (17.2%)	​
Sociodemographic characteristics					​	​
Age at diagnosis	​	​	​	​	​	<0.001
20–29 years	3,664 (4.6%)	2,653 (72.4%)	221 (6%)	93 (2.5%)	697 (19%)	​
30–39 years	17,010 (21.5%)	12,539 (73.7%)	1,132 (6.7%)	482 (2.8%)	2,857 (16.8%)	​
40–49 years	58,416 (73.9%)	42,484 (72.7%)	4,372 (7.5%)	1,706 (2.9%)	9,854 (16.9%)	​
Marital status	​	​	​	​	​	<0.001
Married	35,417 (44.8%)	25,905 (73.1%)	2,448 (6.9%)	790 (2.2%)	6,274 (17.7%)	​
Unmarried	24,070 (30.4%)	16,683 (69.3%)	1,887 (7.8%)	839 (3.5%)	4,661 (19.4%)	​
Unknown	19,603 (24.8%)	15,088 (77%)	1,390 (7.1%)	652 (3.3%)	2,473 (12.6%)	​
SES level	​	​	​	​	​	<0.001
Low	14,421 (18.2%)	10,392 (72.1%)	1,199 (8.3%)	569 (4%)	2,261 (15.7%)	​
Medium	44,973 (56.9%)	32,652 (72.6%)	3,264 (7.3%)	1,291 (2.9%)	7,766 (17.3%)	​
High	19,696 (24.9%)	14,632 (74.3%)	1,262 (6.4%)	421 (2.1%)	3,381 (17.2%)	​
Rurality	​	​	​	​	​	<0.001
All urban	52,611 (66.5%)	37,043 (70.4%)	3,915 (7.4%)	1,617 (3.1%)	10,036 (19.1%)	​
Mostly urban	16,259 (20.6%)	12,890 (79.3%)	1,162 (7.2%)	435 (2.7%)	1,772 (10.9%)	​
Mostly rural	5,567 (7%)	4,182 (75.1%)	344 (6.2%)	115 (2.1%)	926 (16.6%)	​
All rural	4,653 (5.9%)	3,561 (76.5%)	304 (6.5%)	114 (2.5%)	674 (14.5%)	​
Tumor characteristics					​	​
Stage	​	​	​	​	​	<0.001
Localized	19,650 (24.9%)	14,308 (72.8%)	1,053 (5.4%)	462 (2.4%)	3,827 (19.5%)	​
Regionalized	25,168 (31.8%)	18,992 (75.5%)	1,813 (7.2%)	656 (2.6%)	3,707 (14.7%)	​
Distant	14,514 (18.4%)	11,558 (79.6%)	1,096 (7.6%)	487 (3.4%)	1,373 (9.5%)	​
Unknown	19,758 (25%)	12,818 (64.9%)	1,763 (8.9%)	676 (3.4%)	4,501 (22.8%)	​
Grade	​	​	​	​	​	<0.001
Well differentiated	5,511 (7%)	3,856 (70%)	307 (5.6%)	189 (3.4%)	1,159 (21%)	​
Moderately differentiated	36,195 (45.8%)	27,347 (75.6%)	2,499 (6.9%)	931 (2.6%)	5,418 (15%)	​
Poorly differentiated	9,167 (11.6%)	7,068 (77.1%)	509 (5.6%)	219 (2.4%)	1,371 (15%)	​
Undifferentiated	1,357 (1.7%)	1,128 (83.1%)	81 (6%)	28 (2.1%)	120 (8.8%)	​
Unknown	26,860 (34%)	18,277 (68.1%)	2,329 (8.7%)	914 (3.4%)	5,340 (19.9%)	​
Primary site^a^	​	​	​	​	​	<0.001
Left	60,391 (76.4%)	42,678 (70.7%)	4,888 (8.1%)	1,966 (3.3%)	10,859 (18%)	​
Right	18,699 (23.6%)	14,998 (80.2%)	837 (4.5%)	315 (1.7%)	2,549 (13.6%)	​
Year of diagnosis	​	​	​	​	​	<0.001
2006–2010	25,251 (31.9%)	18,837 (74.6%)	1,404 (5.6%)	579 (2.3%)	4,431 (17.6%)	​
2011–2015	25,979 (32.9%)	19,136 (73.7%)	1,725 (6.6%)	729 (2.8%)	4,389 (16.9%)	​
2016–2020	27,860 (35.2%)	19,703 (70.7%)	2,596 (9.3%)	973 (3.5%)	4,588 (16.5%)	​

a Right: cecum to transverse; left: splenic flexure to rectum.

## Results

The majority of patients with EOCRC were male (53.2%), White (54.7%), had timely treatment (72.9%), were aged 40 to 49 years (73.9%), were married (44.8%), lived in medium SES areas (56.9%) and in all urban areas (66.5%), were diagnosed with local (24.9%) or regionalized stage (31.8%), were diagnosed with moderately differentiated tumor (45.8%), had left-sided colorectal cancer (76.4%), and were diagnosed with colorectal cancer during 2016 to 2020 (35.2%; Table 1). A higher proportion of females received timely treatment compared with males (73.1% vs. 72.8%), whereas treatment delays were more common among males (7.5% vs. 6.9%; *P* value < 0.001). Furthermore, racial minority patients were significantly more likely than White patients to experience delays in treatment or to receive no or unknown timing treatment (*P* value < 0.001). Our supplementary analysis also demonstrated similar characteristics when excluding those without or unknown treatment timeliness (Supplementary Table S1).

**Figure 1.**
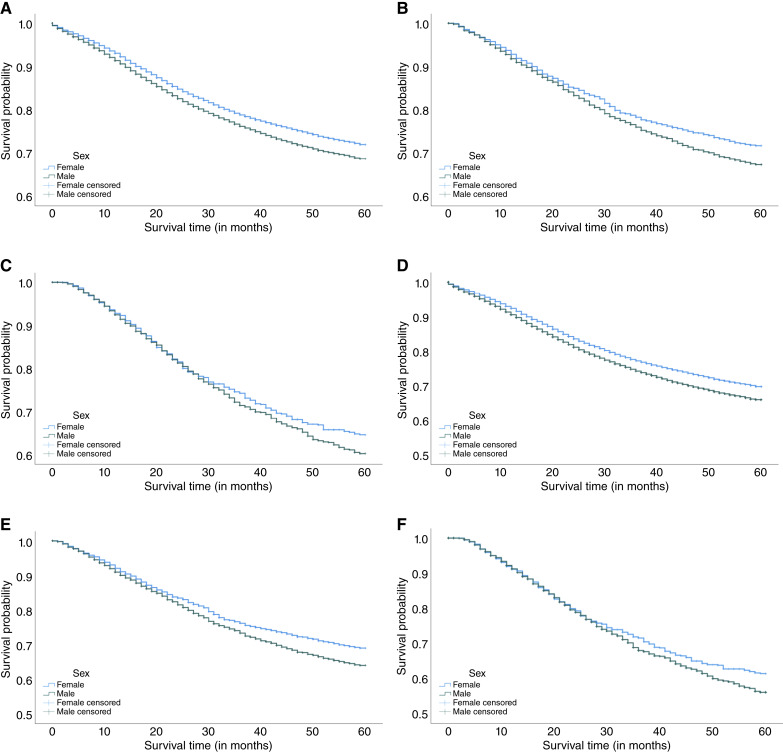
Kaplan–Meier curves for colorectal cancer CSS and OS by sex and timeliness of treatment. **A,** Risk of colorectal cancer death: timely (*P* value < 0.001). **B,** Risk of colorectal cancer death: delayed (*P* value = 0.005). **C,** Risk of colorectal cancer death: severely delayed (*P* value = 0.167). **D,** Risk of death: timely (*P* value < 0.001). **E,** Risk of death: delayed (*P* value = 0.002). **F,** Risk of death: severely delayed (*P* value = 0.091).

**Figure 2.**
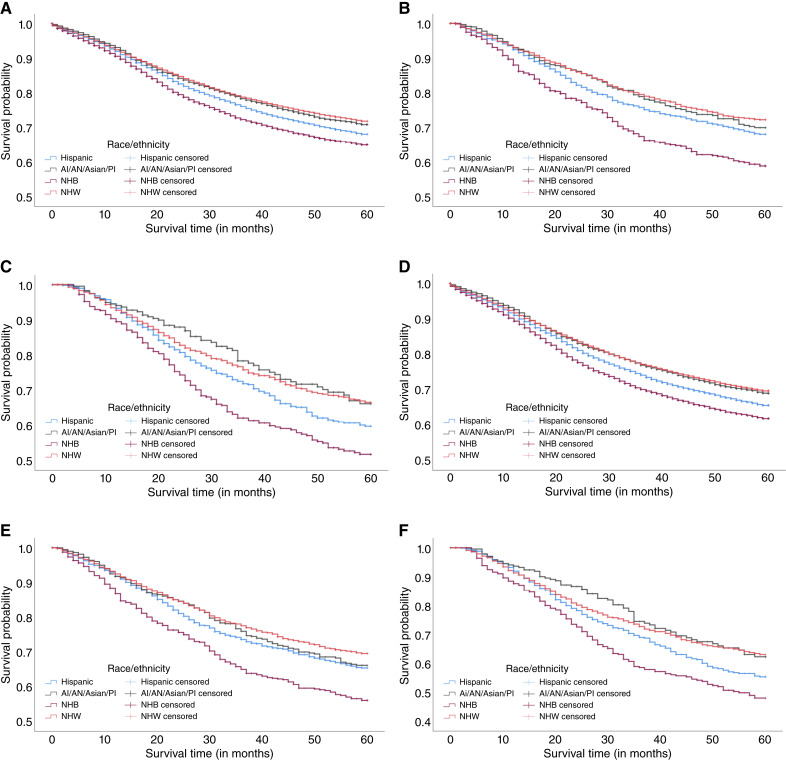
Kaplan–Meier curves for colorectal cancer CSS and OS by race/ethnicity and timeliness of treatment. **A,** Risk of colorectal cancer death: timely (*P* value < 0.001). **B,** Risk of colorectal cancer death: delayed (*P* value < 0.001). **C,** Risk of colorectal cancer death: severely delayed (*P* value < 0.001). **D,** Risk of death: timely (*P* value < 0.001). **E,** Risk of death: delayed (*P* value < 0.001). **F,** Risk of death: severely delayed (*P* value < 0.001).

As shown in Fig. 1A–F, male patients demonstrated lower colorectal cancer–related 5-year survival rates compared with female patients for all timeliness categories (timely, 74% vs. 76.7%, *P* value < 0.001; delayed, 75.3% vs. 78.1%, *P* value = 0.167; and severely delayed, 69.5% vs. 73.3%, *P* value = 0.002). The same was true for all-cause 5-year survival rates (timely, 71.3% vs. 74.5%, *P* value = 0.005; delayed, 72.2% vs. 75.7%, *P* value < 0.001; and severely delayed, 65.2% vs. 70%, *P* value = 0.091). No statistically significant differences were found in colorectal cancer–related survival rates for delayed treatment or in all-cause 5-year survival rates with severely delayed treatment. Furthermore, in Fig. 2A–F, NHB patients consistently showed the lowest survival for colorectal cancer–related survival rates (timely, 70.4%; delayed, 67.4%; and severely delayed, 60.8%) and all-cause survival rates (timely, 67%; delayed, 64.2%; and severely delayed, 65.5%) than patients of other races regardless of treatment timeliness. Finally, we used the Kaplan–Meier approach to evaluate the Cox PH assumption in our three exposures of interest, separately. The survival curves showed similar trends and generally did not cross, supporting the PH assumption (19).

### Multivariable analysis

As shown in Table 2, adjusted analysis indicated that male patients had 15% higher risk of dying from colorectal cancer (HR, 1.15; 95% CI, 1.12–1.19) and 16% higher risk of death from any causes (HR, 1.16; 95% CI, 1.13–1.20) compared with females. Compared with NHW patients, NHB, Hispanic, and AI/AN/Asian/PI patients were 24% (HR, 1.24; 95% CI, 1.19–1.30), 7% (HR, 1.07; 95% CI, 1.03–1.12), and 6% (HR, 1.06; 95% CI, 1–1.12) more likely to die from colorectal cancer. A similar pattern was observed for all-cause mortality: NHB, Hispanic, and AI/AN/Asian/PI patients were 24%, 7%, and 6% more likely to die from any causes, respectively. With regard to timeliness of treatment, patients with EOCRC experiencing severe delays in treatment initiation were more likely to die from colorectal cancer (HR, 1.09; 95% CI, 1–1.18) and any causes (HR, 1.12; 95% CI, 1.04–1.21) by 9% to 12% than those receiving treatment promptly. Finally, patients with no or unknown treatment time were 69% (HR, 0.31; 95% CI, 0.29–0.33) and 68% (HR, 0.32; 95% CI, 0.30–0.33) less likely to die from colorectal cancer and any causes than those who received timely treatment, respectively. Finally, interactions between sex and timeliness of treatment and race/ethnicity and timeliness of treatment were not observed in base and adjusted models but identifying demographic groups and understanding the impact of timeliness of treatment on mortality remain critical for developing targeted interventions. Our supplementary analysis also demonstrated similar results when excluding the no/unknown group (Supplementary Table S2) and using the competing risk model (Supplementary Table S3).

**Table 2.** Association between sex, race, and timeliness of treatment on survival.

​	CSS		OS	
	Base model	Adjusted model	Base model	Adjusted model
	HR (95% CI)			
Sex	​	​	​	​
Female	Reference	Reference	Reference	Reference
Male	***1.17 (1.13–1.20)***	***1.15 (1.12–1.19)***	***1.18 (1.14–1.21)***	***1.16 (1.13–1.20)***
Race/ethnicity	​	​	​	​
NHW	Reference	Reference	Reference	Reference
NHB	***1.37 (1.31–1.43)***	***1.24 (1.19–1.30)***	***1.39 (1.33–1.44)***	***1.24 (1.19–1.29)***
Hispanic	***1.17 (1.13–1.21)***	***1.07 (1.03–1.12)***	***1.18 (1.13–1.22)***	***1.07 (1.03–1.12)***
AI/AN/Asian/PI^a^	1.03 (0.98–1.09)	***1.06 (1–1.12)***	1.03 (0.97–1.08)	***1.06 (1.01–1.12)***
Timeliness of treatment				
Timely	Reference	Reference	Reference	Reference
Delayed	1.02 (0.96–1.07)	0.96 (0.91–1.02)	1.03 (0.98–1.09)	0.98 (0.93–1.03)
Severely delayed	***1.24 (1.15***–***1.34)***	***1.09 (1***–***1.18)***	***1.28 (1.19***–***1.37)***	***1.12 (1.04***–***1.21)***
No/unknown	***0.30 (0.28***–***0.32)***	***0.31 (0.29***–***0.33)***	***0.30 (0.29***–***0.32)***	***0.32 (0.30***–***0.33)***
*P* value for sex* timeliness of treatment	0.965	0.958	0.954	0.985
*P* value for race* timeliness of treatment	0.136	0.771	0.254	0.626

Notes: Adjusted models included sociodemographic characteristics, tumor characteristics, and year of diagnosis.

Bold and italicized text indicates statistically significant results.

a AI/AN/Asian/PI were combined in models because of small sample size.

In Table 3, male patients consistently exhibited a higher risk of mortality from colorectal cancer and all causes, with HRs indicating a 14% to 15% increased risk across both timely and delayed treatment initiation. Specifically, colorectal cancer mortality was elevated among males compared with females across all treatment timing except severely delayed: timely (HR, 1.15; 95% CI, 1.12–1.19), delayed treatment (HR, 1.14; 95% CI, 1.02–1.27), and no or unknown treatment (HR, 1.18; 95% CI, 1.05–1.33). Similarly, for overall mortality, the greater risk of death was observed across treatment timeliness (timely: HR, 1.16; 95% CI, 1.13–1.20; delayed: HR, 1.15; 95% CI, 1.04–1.28; and no/unknown: HR, 1.20; 95% CI, 1.07–1.34). Among NHB patients with EOCRC, regardless of timeliness of treatment, those with colorectal cancer were more likely to die from colorectal cancer by 23% to 37% and any causes by 21% to 34%. Compared with NHW patients, Hispanic patients who received timey treatment were also more likely to die from colorectal cancer (HR, 1.08; 95% CI, 1.03–1.13) and any causes (HR, 1.08; 95% CI, 1.03–1.12) by 8%. They were also more likely to die from colorectal cancer by 22% (HR, 1.22; 95% CI, 1.03–1.44) and any causes by 23% (HR, 1.23; 95% CI, 1.06–1.44) when receiving no treatment or unknown treatment status. Finally, AI/AN/Asian/PI individuals experienced 19% increased risk of overall mortality when receiving delayed treatment (HR, 1.19; 95% CI, 1.00–1.42).

**Table 3.** Association between sex and race on survival by timeliness of treatment.

​	CSS				OS			
	Timely	Delayed	Severely delayed	No/unknown	Timely	Delayed	Severely delayed	No/unknown
	HR (95% CI)^a^							
Sex	​	​	​	​	​	​	​	​
Female	Reference	Reference	Reference	Reference	Reference	Reference	Reference	Reference
Male	***1.15 (1.12***–***1.19)***	***1.14 (1.02***–***1.27)***	1.13 (0.96–1.32)	***1.18 (1.05***–***1.33)***	***1.16 (1.13***–***1.20)***	***1.15 (1.04***–***1.28)***	1.14 (0.99–1.33)	***1.20 (1.07***–***1.34)***
Race	​	​	​	​	​	​	​	​
NHW	Reference	Reference	Reference	Reference	Reference	Reference	Reference	Reference
NHB	***1.23 (1.17***–***1.29)***	***1.37 (1.17***–***1.61****)*	***1.24 (1***–***1.54****)*	***1.33 (1.13***–***1.58)***	***1.22 (1.17***–***1.28)***	***1.34 (1.15***–***1.57)***	1.21 (0.99–1.49)	***1.32 (1.12***–***1.54)***
Hispanic	***1.08 (1.03***–***1.13)***	1.03 (0.89–1.19)	0.95 (0.78–1.17)	***1.22 (1.03***–***1.44)***	***1.08 (1.03***–***1.12)***	1.02 (0.89–1.17)	0.97 (0.80–1.17)	***1.23 (1.06***–***1.44)***
AI/AN/Asian/PI^b^	***1.06 (1***–***1.13)***	1.14 (0.94–1.38)	0.95 (0.71–1.26)	1.33 (0.82–1.29)	1.05 (0.99–1.12)	***1.19 (1***–***1.42)***	0.98 (0.75–1.27)	1.07 (0.87–1.33)

Bold and italicized text indicates statistically significant results.

a All models were adjusted for sociodemographic characteristics, tumor characteristics, and year of diagnosis.

b AI/AN/Asian/PI were combined in models because of small sample size.

## Discussion

This is the first integrated study examining the impact of specific demographic groups on CSS (i.e., cancer related) and OS (i.e., from any cause) for patients with EOCRC, while accounting for treatment timeliness, using a nationally representative sample from the United States. Two key findings emerge: (i) male and NHB patients consistently exhibited poorer survival outcomes, regardless of whether treatment was timely or delayed. This pattern was not evident for NHB patients with severely delayed treatment in terms of OS and (ii) Hispanic patients with no or unknown treatment and AI/AN/Asian/PI patients who experienced delayed treatment faced an increased risk of mortality.

First, we found that male patients consistently exhibited a higher risk of mortality from colorectal cancer and from any cause, whether treatment was initiated on a timely basis, delayed by any length of time, or not received or no information on timelines available. Consistent with a prior study, male patients with EOCRC show a worse OS by 18% and CSS by 14% (17). Also aligning with our results, a prior study reported that male patients with colorectal cancer had a 43% higher overall risk of mortality than female patients (20). In our study, male patients also demonstrated the lower 5-year survival for colorectal cancer despite receiving timely treatment initiation for CSS (74% vs. 77% in females) and OS (71.3% vs. 74.5% in females), which is in line with literature (17). Afify and colleagues (17) reported that males had lower 5-year OS (65.7% vs. 69.9%) and CSS (67.9% vs. 71.5%) compared with females. However, Kaplan–Meier curves provided unadjusted survival estimates, whereas the Cox PH models adjusted for potential confounders. Therefore, our results from the adjusted models offer a more accurate assessment of the effect of treatment timeliness on survival. Factors that may contribute to poorer survival outcomes among males regardless of treatment timeliness include cultural norms such as stoicism and self-reliance among male patients (21–23). Prior studies reported that males were 38% less likely to participate in routine health care compared with females, which may lead to diagnostic delays and, in turn, help explain worse survival outcomes (24). However, we were unable to evaluate the impact of cultural norms in this analysis as this information is not collected in SEER; this remains an area for future research. Other factors such as biological differences (e.g., hormonal and genetic), response to treatment (e.g., chemotherapy tolerance), and psychosocial influences may also contribute to the observed survival disparity for young males (25, 26). Future research should explore these sex-specific determinants to inform tailored strategies that improve early detection and outcomes for young men with EOCRC.

Another important finding is that NHB patients experienced worse survival outcomes regardless of treatment timeliness, with highest estimates observed in those with delayed treatment by 37% for CSS and 34% for OS. Consistent with prior literature, younger NHB patients typically experience a 42% greater risk of cancer mortality compared with NHW patients (16). They also face substantially higher colorectal cancer–specific mortality risks—69% for stage II and 98% for stage III colon cancers (15). Treatment delays may partly explain these poorer survival outcomes (9). For example, Black patients are significantly more likely to experience treatment delays—over 60 days by 52% and over 90 days by 38% (7). Although this prior literature does not examine survival directly, such delays can lead to delayed or reduced receipt of guideline-concordant care, which has been associated with poorer survival outcomes (8). These systemic delays may therefore contribute to the worse survival observed in our study. In addition, NHB patients with EOCRC also consistently showed the lowest survival despite receiving timely care (CSS: 70.4%; OS: 67%) in our analysis. Similar to findings in male patients, treatment timeliness may not be the primary determinant of survival outcomes for NHB patients. Including treatment timeliness in our analysis provides a more nuanced understanding of access to care. Although previous studies often assumed delays or receiving no treatment contributed to survival gaps, our results show that disparities remain even among those with timely treatment initiation, suggesting that other factors, such as structural barriers (e.g., access to quality of care or underinsured) or medical mistrust, may also play a significant role in mortality for NHB patients (27–29). Furthermore, the presence of comorbidities may partly explain the higher mortality observed among NHB patients (30). Despite various contributing factors, research focusing on young NHB populations considering treatment timeliness remains limited. Further investigation is needed to identify and understand the specific barriers driving these disparities.

Furthermore, prior evidence also suggested that Hispanic patients with colorectal cancer were associated with delay in treatment by 27% for >60 days and 17% for >90 days (7), which may have potential to explain worse survival. However, our findings revealed a contrasting pattern among patients with EOCRC. Specifically, Hispanic patients were 8% more likely to die from colorectal cancer and any causes despite receiving timely treatment. This divergence from prior research may be attributed to the fact that earlier study primarily focused on the general colorectal cancer population, whereas our analysis targeted patients with EOCRC, who may face distinct clinical and sociodemographic challenges. For example, comorbidities such as diabetes and hypertension are common among Hispanic populations and may complicate the treatment process, potentially affecting survival outcomes (31). However, we were unable to assess the presence of comorbidities on survival outcomes because of the lack of available information.

We also found that Hispanic patients were 22% to 23% more likely to die from colorectal cancer and any causes when receiving no treatment or unknown treatment status. In a California study, Hispanic patients with colorectal cancer were more likely to be undertreated and also more likely to have treatment delays longer than 60 days by 27% (7), both of which may contribute to increased mortality risk. Similar barriers with delays in treatment, our finding may reflect disparities in care access, documentation gaps, or systemic barriers (e.g., transportation challenges) disproportionately affecting these populations, particularly for racial minorities (14). Social determinants of health (SDoH), including SES, insurance status, and cultural or language barriers, could contribute to no access to treatment (11, 32). Furthermore, the “no/unknown treatment time” group may include patients who received treatment promptly but lacked complete documentation (33) as missing data often reflect administrative gaps rather than actual delays. Future studies should investigate whether these patterns represent true treatment delays or data limitations and explore targeted interventions to reduce inequities for these groups.

Finally, AI/AN/Asian/PI patients were also more likely to die from any causes by 19% when receiving delayed treatment in our study. This is in line with a recent EOCRC study, in which they found that racial minorities (e.g., Native Hawaiian or other PI patients) were 34% more likely to die from colorectal cancer (34). However, this research has shown that receiving guideline-concordant treatment was still associated with a 33% higher risk of colorectal cancer mortality among these racial minorities (34). The discrepancy between these findings and ours may be due to differences in study populations. The referenced study focused on patients in California, whereas our analysis included a more diverse population across 22 SEER registries nationwide. Another possible explanation for our findings is that racial minorities often live in socioeconomically disadvantaged areas which can limit access to high-quality care and create structural barriers, including low health literacy and limited support systems (35). These factors can adversely affect cancer outcomes.

### Implications

Our findings emphasize that improving outcomes requires more than ensuring timely treatment. For male and NHB patients, structural and cultural barriers—such as systemic bias, socioeconomic challenges, and mistrust in healthcare—may continue to limit equitable care (27–29). Addressing these issues calls for targeted interventions that incorporate culturally responsive practices, community engagement, and provider training to reduce bias. At the same time, AI, AN, Asian, and PI patients face persistent gaps in access to timely care, which can be mitigated through strategies like improving geographic and financial accessibility and tailoring services to linguistic and cultural needs (36). Collectively, these findings underscore the importance of equity-driven approaches that go beyond treatment timeliness to dismantle systemic barriers and ensure inclusive, high-quality care for all populations.

### Strengths and limitations

The main strength of this study is the categorization of treatment timeliness into distinct groups: timely, delayed, severely delayed, and no/unknown. This framework enables a more nuanced analysis of how varying degrees of delay affect survival outcomes and helps identify specific patient populations that may be disproportionately affected. Despite the strengths of this study, some limitations should be acknowledged. First, several important SDoH (e.g., structural barriers, financial strain, and awareness of colorectal cancer risk) could not be assessed as SEER does not collect these factors. Similarly, key demographic factors such as income, education level, and health insurance status are also not available in cancer registry data despite their well-established links to cancer care access and outcomes as part of SDoH. Furthermore, the lack of day-level treatment timing data—as SEER reports time in months—limits the precision of our analysis. This is particularly relevant given that the majority of patients (73%) initiated treatment within 1 month. Furthermore, SEER does not comprehensively capture all treatment modalities, which may also lead to misclassification of treatment timeliness if initial treatments were not recorded. Future research utilizing more granular and integrated clinical data may help to address the limitations and provide a clearer understanding of the observed disparities. Third, the SEER program’s categorization of AI/AN individuals as a single group, and the grouping of Asian/PI populations, prevented disaggregation. This limitation prevents us from examining within-group heterogeneity and restricts our ability to identify the distinct challenges and healthcare interactions experienced by specific racial and ethnic subpopulations as each group may face unique barriers to cancer care and may experience different impacts on cancer outcomes. Finally, incomplete or inconsistent reporting of treatment initiation timelines from SEER program may contribute to an underestimation of disparities. High levels of missingness could introduce bias and reduce the precision of our estimates as individuals with incomplete data may differ systematically from those with complete information. Therefore, our findings should be interpreted with caution. To address this limitation, we conducted additional analyses by excluding individuals with missing or unknown treatment timing data and examining demographic characteristics for those with unknown information. The demographic characteristics and association remain similar, suggesting that the impact of incomplete or inconsistent treatment information is likely minimal. Nonetheless, future research incorporating more comprehensive treatment data could further reinforce these conclusions.

### Conclusion

Male and NHB patients exhibited poorer survival outcomes regardless of whether treatment was timely or delayed. Hispanic patients who received no or unknown treatment, as well as AI/AN/Asian/PI patients who experienced delayed treatment, were also more likely to face increased mortality risk. These findings underscore the need for interventions beyond timely treatment, particularly for male and NHB patients. Targeted strategies should focus on improving access to high quality of care for AI/AN/Asian/PI patients and addressing structural and cultural barriers for male and NHB patients.

## Supplementary Material

Supplementary Figure 1 Sample selection flowchart

Supplementary Table 1 Patient Characteristics by Treatment Time Inclusion

Supplementary Table 2 Survival by Sex, Race/Ethnicity, and Timely Treatment

Supplementary Table 3 sex, race, and timeliness of treatment on CRC mortality

## Data Availability

Data analyzed in this study are available via the SEER Program registry (https://seer.cancer.gov/). Downstream analytic data are available upon request from the corresponding author.
