# Supplementary material for: Treatment Delays and Survival Divides: Race, Sex, and Early-Onset Colorectal Cancer Disparities
Source: Cancer Res Commun. 2026 Jan 29;6(1):235–44. doi: 10.1158/2767-9764.CRC-25-0659 (PMC12853325; doi:10.1158/2767-9764.CRC-25-0659)
Supplement: Supplementary Figure 1 — Sample selection flowchart [file crc-25-0659_supplementary_figure_1_suppsf1.pdf]

Supplementary Figure 1.

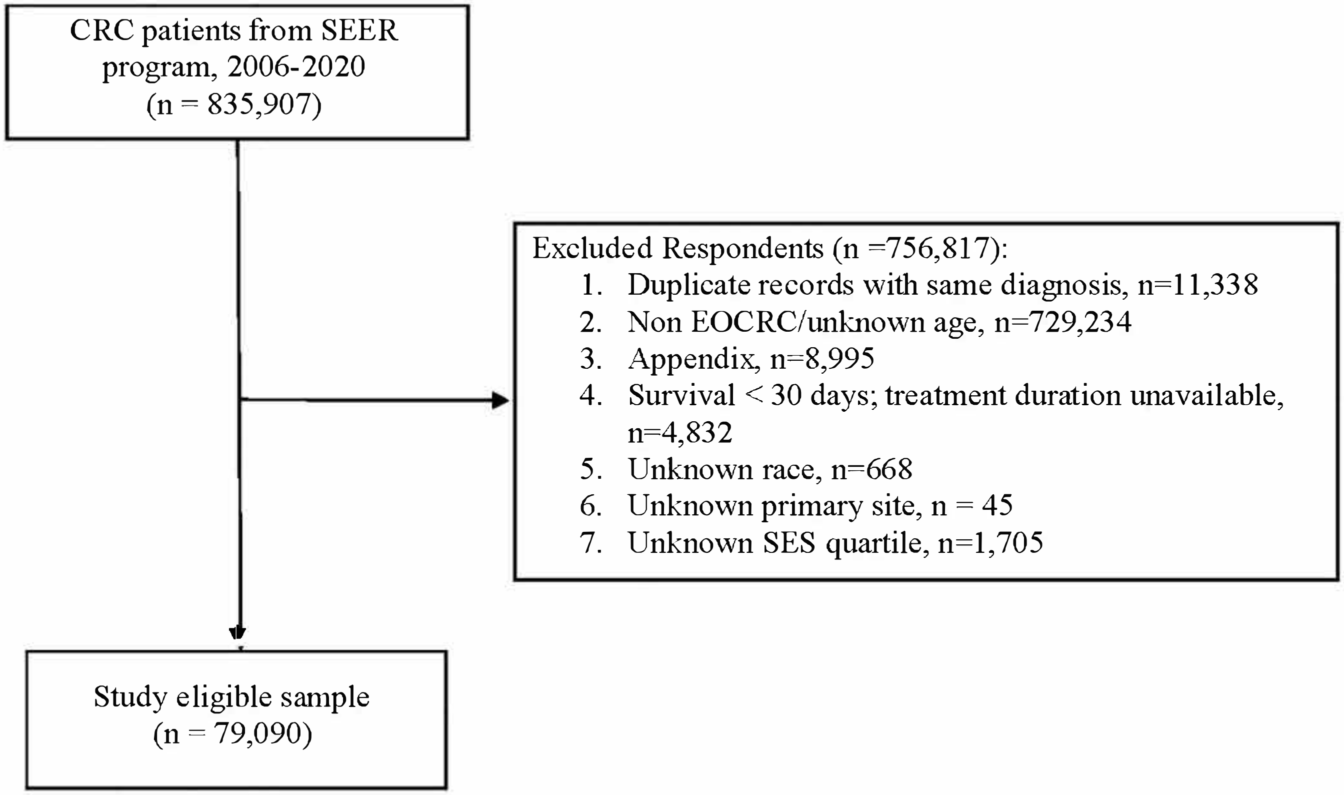

Supplementary Figure 1. Consort diagram showing the derivation of the analytic sample.
