# Supplementary material for: Treatment Delays and Survival Divides: Race, Sex, and Early-Onset Colorectal Cancer Disparities
Source: Cancer Res Commun. 2026 Jan 29;6(1):235–44. doi: 10.1158/2767-9764.CRC-25-0659 (PMC12853325; doi:10.1158/2767-9764.CRC-25-0659)
Supplement: Supplementary Table 1 — Patient Characteristics by Treatment Time Inclusion [file crc-25-0659_supplementary_table_1_suppst1.docx]

**Supplementary Table 1. EOCRC Patient Characteristics by Treatment Time Inclusion**

|  | **Including No/Unknown time**  **(n=79,090)** | **Excluding No/Unknown time**  **(n=65,682)** |
| --- | --- | --- |
| **Sex** |  |  |
| Female | 36,998 (46.8%) | 30,591 (46.6%) |
| Male | 42,092 (53.2%) | 35,091 (53.4%) |
| **Race/Ethnicity** |  |  |
| White | 43,283 (54.7%) | 35,835 (54.6%) |
| Black | 10,915 (13.8%) | 8,836 (13.5%) |
| Hispanic | 17,245 (21.8%) | 14,641 (22.3%) |
| AI/AN | 510 (0.6%) | 457 (0.7%) |
| Asian/PI | 7,137 (9.0%) | 5,913 (9.0%) |
| **Sociodemographic characteristics** |  |  |
| **Age at diagnosis** |  |  |
| 20-29 years | 3,664 (4.6%) | 2,967 (4.5%) |
| 30-39 years | 17,010 (21.5%) | 14,153 (21.6%) |
| 40-49 years | 58,416 (73.9%) | 48,562 (73.9%) |
| **Marital status** |  |  |
| Married | 35,417 (44.8%) | 29,143 (44.4%) |
| Unmarried | 24,070 (30.4%) | 19,409 (29.6%) |
| Unknown | 19,603 (24.8%) | 17,130 (26.1%) |
| **SES level** |  |  |
| Low | 14,421 (18.2%) | 12,160 (18.5%) |
| Medium | 44,973 (56.9%) | 37,207 (56.7%) |
| High | 19,696 (24.9%) | 16,315 (24.8%) |
| **Rurality** |  |  |
| All urban | 52,611 (66.5%) | 42,575 (64.8%) |
| Mostly urban | 16,259 (20.6%) | 14,487 (22.1%) |
| Mostly rural | 5,567 (7.0%) | 4,641 (7.1%) |
| All rural | 4,653 (5.9%) | 3,979 (6.1%) |
| **Tumor characteristics** |  |  |
| **Stage** |  |  |
| Localized | 19,650 (24.9%) | 15,823 (24.1%) |
| Regionalized | 25,168 (31.8%) | 21,461 (32.7%) |
| Distant | 14,514 (18.4%) | 13,141 (20.0%) |
| Unknown | 19,758 (25.0%) | 15,257 (23.2%) |
| **Grade** |  |  |
| Well differentiated | 5,511 (7.0%) | 4,352 (6.6%) |
| Moderately differentiated | 36,195 (45.8%) | 30,777 (46.9%) |
| Poorly differentiated | 9,167 (11.6%) | 7,796 (11.9%) |
| Undifferentiated | 1,357 (1.7%) | 1,237 (1.9%) |
| Unknown | 26,860 (34.0%) | 21,520 (32.8%) |
| **Primary site** ^a^ |  |  |
| Left | 60,391 (76.4%) | 49,532 (75.4%) |
| Right | 18,699 (23.6%) | 16,150 (24.6%) |
| **Year of diagnosis** |  |  |
| 2006-2010 | 25,251 (31.9%) | 20,820 (31.7%) |
| 2011-2015 | 25,979 (32.9%) | 21,590 (32.9%) |
| 2016-2020 | 27,860 (35.2%) | 23,272 (35.4%) |

Abbreviations: EOCRC, early-onset colorectal cancer; NHW, non-Hispanic White; NHB, non-Hispanic Black; AI/AN, American Indian/Alaska Native; PI, pacific islander; SES, socioeconomic status.

^a^ Right: cecum to transverse; left: splenic flexure to rectum.
