# Supplementary material for: Treatment Delays and Survival Divides: Race, Sex, and Early-Onset Colorectal Cancer Disparities
Source: Cancer Res Commun. 2026 Jan 29;6(1):235–44. doi: 10.1158/2767-9764.CRC-25-0659 (PMC12853325; doi:10.1158/2767-9764.CRC-25-0659)
Supplement: Supplementary Table 2 — Survival by Sex, Race/Ethnicity, and Timely Treatment [file crc-25-0659_supplementary_table_2_suppst2.docx]

**Supplementary Table 2. Survival by Sex, Race/Ethnicity, and Timely Treatment (Excluding No/Unknown) (n=65,682)**

|  | **Causes-specific survival** | | **All cause survival** | |
| --- | --- | --- | --- | --- |
|  | **Unadjusted model** | **Adjusted model** | **Unadjusted model** | **Adjusted model** |
|  | **HR (95%CI)** | | | |
| **Sex** |  |  |  |  |
| Female | Reference | Reference | Reference | Reference |
| Male | ***1.16 (1.13,1.20)*** | ***1.15 (1.12, 1.19)*** | ***1.17 (1.14, 1.21)*** | ***1.16(1.13, 1.20)*** |
| **Race/Ethnicity** |  |  |  |  |
| White | Reference | Reference | Reference | Reference |
| Black | ***1.37(1.31, 1.43)*** | ***1.24(1.18, 1.29)*** | ***1.39(1.33, 1.45)*** | ***1.23(1.18, 1.29)*** |
| Hispanic | ***1.16(1.12, 1.21)*** | ***1.07(1.02, 1.11)*** | ***1.17(1.12, 1.21)*** | ***1.06(1.02, 1.11)*** |
| AI/AN/Asian/PI | 1.03(0.98, 1.09) | ***1.06(1.01, 1.13)*** | 1.02(0.97, 1.08) | ***1.06(1.01, 1.12)*** |
| **Timeliness Treatment** | | | | |
| Timely | Reference | Reference | Reference | Reference |
| Delayed | 1.01(0.96, 1.07) | 0.96(0.91, 1.02) | 1.03(0.97, 1.08) | 0.98(0.93, 1.03) |
| Severely Delayed | ***1.23(1.14, 1.33)*** | ***1.08(1.00, 1.17)*** | ***1.27(1.18, 1.37)*** | ***1.12(1.04, 1.20)*** |
| **P-value for Sex* Timeliness Treatment** | 0.889 | 0.896 | 0.901 | 0.935 |
| **P-value for Race* Timeliness Treatment** | 0.048 | 0.590 | 0.119 | 0.436 |

Abbreviations: HR, hazard ratio; NHW, non-Hispanic White; NHB, non-Hispanic Black; AI/AN, American Indian/Alaska Native; PI, pacific islander.

Bold and Italicized text indicates statistically significant results.
