# Supplementary material for: Treatment Delays and Survival Divides: Race, Sex, and Early-Onset Colorectal Cancer Disparities
Source: Cancer Res Commun. 2026 Jan 29;6(1):235–44. doi: 10.1158/2767-9764.CRC-25-0659 (PMC12853325; doi:10.1158/2767-9764.CRC-25-0659)
Supplement: Supplementary Table 3 — sex, race, and timeliness of treatment on CRC mortality [file crc-25-0659_supplementary_table_3_suppst3.docx]

**Supplementary Table 3. Association between sex, race, and timeliness of treatment on CRC mortality using competing risk model**

|  | **Causes-specific survival** | |
| --- | --- | --- |
|  | **Unadjusted model** | **Adjusted model** |
|  | **HR (95% CI)** | |
| **Sex** |  |  |
| Female | Reference | Reference |
| Male | ***1.17 (1.13,1.20)*** | ***1.16 (1.12, 1.19)*** |
| **Race/Ethnicity** |  |  |
| White | Reference | Reference |
| Black | ***1.31(1.26, 1.36)*** | ***1.21(1.16, 1.27)*** |
| Hispanic | ***1.14(1.10, 1.18)*** | ***1.06(1.02, 1.10)*** |
| AI/AN/Asian/PI ^a^ | 1.03(0.98, 1.08) | ***1.06(1.00, 1.12)*** |
| **Timeliness of Treatment** |  |  |
| Timely | Reference | Reference |
| Delayed | 1.03(0.98, 1.09) | 0.97(0.92, 1.03) |
| Severely Delayed | ***1.25(1.16, 1.34)*** | ***1.10(1.02, 1.19)*** |
| No/Unknown | ***0.39(0.37,0.41)*** | ***0.41(0.39,0.43)*** |

Abbreviations: CRC, colorectal cancer; HR, hazard ratio; NHW, non-Hispanic White; NHB, non-Hispanic Black; AI/AN, American Indian/Alaska Native; PI, pacific islander.

Bold and Italicized text indicates statistically significant results.

^a^ AI/AN/Asian/PI were combined in models due to small sample size.
